# TC-HUR: A Tri-Phase Cauchy-Assisted Hunger Games Search and Unified Runge–Kutta Optimizer for Robust DNA Data Storage

**DOI:** 10.3390/ijms27073134

**Published:** 2026-03-30

**Authors:** Beyza Öztürk, Ayşenur İgit, Aylin Kaya, Zeynep Tuğsem Çamlıca, Selen Arıcı, Muhammed Faruk Şahin

**Affiliations:** 1 Department of Electrical-Electronics Engineering, Faculty of Engineering and Natural Sciences, Istanbul Atlas University, 34408 Istanbul, Türkiye; 2 Department of Software Engineering, Faculty of Engineering and Natural Sciences, Istanbul Atlas University, 34408 Istanbul, Türkiye; 3 Department of Molecular Biology and Genetics, Faculty of Engineering and Natural Sciences, Istanbul Atlas University, 34408 Istanbul, Türkiye; 4 Department of Computer Engineering, Faculty of Engineering and Natural Sciences, Istanbul Atlas University, 34408 Istanbul, Türkiye

**Keywords:** DNA data storage, metaheuristic optimization, biophysical constraints, high-dimensional sequence design, error-aware encoding

## Abstract

Although DNA-based data storage theoretically provides an information density of 2 bits per nucleotide, biochemical constraints transform sequence design into a high-dimensional constrained combinatorial optimization problem. The high computational cost and low encoding efficiency of conventional rule-based approaches make metaheuristic methods an effective alternative. This study proposes the TC-HUR hybrid algorithm to simultaneously optimize information density and conflicting biophysical constraints, including homopolymer (HP) length, GC content, melting temperature ($T_{m}$), and reverse-complement (RC) similarity. The method escapes local optima using Cauchy jump-enhanced Hunger Games Search (HGS), performs high-precision exploitation via Runge–Kutta (RUN) operators, and refines constraint violations at the nucleotide level through an adaptive intensive mutation mechanism. The algorithm is evaluated on a complex dataset of 1853 nucleotides under different noise regimes. TC-HUR outperforms RUN by 2.5% and HGS by 16.7% in average fitness. While maintaining homopolymer length near the ideal threshold, it reduces reverse-complement similarity to 19.10%, ensuring high sequence diversity. Under high-noise conditions, TC-HUR achieves a normalized edit distance of 0.1290, reducing insertion–deletion (indel) errors by approximately 14%. The results demonstrate that the proposed model effectively generates biophysically synthesizable and noise-resilient DNA codes.

## 1. Introduction

In recent years, the acceleration of digitalization processes, together with the increasing density of data and a data production ecosystem dominated by computational methods, has led to an exponential growth in the global volume of digital data [1,2,3]. Currently, these massive data streams are predominantly stored through conventional silicon-based semiconductor storage units. Although such traditional systems provide high access speeds, they exhibit critical bottlenecks in terms of physical storage density limits, material lifespan, and sustainable energy requirements [4,5,6]. The fact that the rate of data generation has surpassed the capacity growth rate of existing storage technologies has, in recent years, driven researchers to explore alternative storage media offering higher information density and long-term stability [7]. In this context, DNA-based data storage systems—characterized by exceptionally high information density at the molecular level, the potential for energy-independent storage, and remarkable chemical stability—have emerged in the literature as a complementary and alternative approach to conventional storage paradigms [8]. DNA-based data storage relies on the principle of mapping digital “0” and “1” sequences into biological nucleotide sequences “A, T, C, G”, subsequently synthesizing these sequences artificially and storing them in vitro, and finally converting them back into digital form using next-generation sequencing technologies when retrieval is required. This four-nucleotide alphabet theoretically enables an information density of 2 bits per nucleotide, offering substantially greater capacity compared to conventional magnetic and optical storage media [9]. However, in practical implementations, this theoretical upper bound cannot be directly achieved due to biochemical and sequence-related constraints associated with DNA synthesis and sequencing processes [10,11]. Requirements such as maintaining GC content balance, limiting homopolymer repetitions, and ensuring sufficient inter-sequence dissimilarity constitute fundamental factors that determine error rates. Consequently, a pronounced discrepancy arises between the theoretical capacity and the effective net data density achievable in practice [12,13,14]. These biochemical and sequence-level constraints directly restrict randomness within the sequence space and lead to a structural gap between theoretical capacity and practical net information density, thereby limiting overall system performance. Existing approaches developed to address these constraints are predominantly shaped around rule-driven and coding-based strategies. Conventional coding approaches developed within this framework are generally structured around two main strategies: biochemical screening-based methods and rule-based coding schemes. Screening-based approaches aim to enhance system reliability by excluding biologically unsuitable sequences during the post-processing stage; however, this process results in increased computational costs and significant losses in coding efficiency. Conversely, rule-based coding schemes reduce the need for screening, but suppress sequence diversity, thereby decreasing primer orthogonality in large-scale DNA pools and increasing the risk of cross-hybridization [15].

The nonlinear trade-off between these two extreme approaches offered by conventional methods transforms DNA sequence design into a multidimensional, discrete, and constrained combinatorial optimization problem. At this point, metaheuristic approaches—whose capability to explore complex search spaces has been well established in the engineering and computer science literature—constitute an effective solution paradigm for overcoming the theoretical and practical bottlenecks of traditional methods [16]. Rather than pursuing exact solutions, these heuristic mechanisms, inspired by biological or physical phenomena, generate solutions that are near the global optimum and operationally feasible under multiple conflicting objectives [17,18]. Metaheuristic optimization holds the potential to harmonize biochemical feasibility, information density, and scalable access requirements within a holistic perspective [19,20]. In recent years, metaheuristic algorithms have emerged as an alternative research direction in the DNA coding literature to mitigate the structural limitations imposed by conventional strategies [21,22]. These approaches aim to analyze the sequence space within a broader projection than deterministic methods by directly integrating parameters such as GC content, homopolymer constraints, and Hamming distance into their objective functions. Nevertheless, the adaptation of these methods to DNA data storage architectures remains limited to a relatively small number of studies. The existing literature exhibits structural deficiencies, particularly with respect to convergence rate and global optimum stability in high-dimensional sequence sets. Furthermore, a considerable portion of current studies focuses primarily on specific constraint sets rather than optimizing the complex correlation between biochemical constraints and sequence entropy through a comprehensive and integrated framework. As research in DNA data storage continues to advance, biological constraints—including GC content imbalance, homopolymer formation, and sequence stability—exert critical influences on storage reliability and long-term data integrity. In order to address these challenges, algorithmic approaches and optimization methods developed within the field of computer science are increasingly being employed. In this context, both traditional constraint-based coding methods and approaches based on metaheuristic optimization algorithms have been applied in the literature to generate DNA sequences with enhanced biological stability. In this section, the related studies that form the theoretical and methodological foundation of the proposed hybrid optimization algorithm are examined under two main categories: traditional methods and metaheuristic-based approaches.

### 1.1. Traditional Approaches for DNA Sequence Design in DNA Data Storage

Increasing information density, managing biochemical constraints, and reducing synthesis-sequencing costs represent the primary research objectives of DNA-based data storage systems. Accordingly, numerous cryptographic, steganographic, and coding-based methods have been developed in the literature. However, these approaches generally produce unavoidable trade-offs among capacity, biological feasibility, and computational cost. Within this scope, a DNA-XOR-based steganographic method has been proposed in order to complement the capacity limitations of the Cas12a-assisted DNA steganography method (CADS) architecture [23]. In the proposed DNA-XOR-based embedding approach, each nucleotide is encoded to correspond to a two-bit binary representation, thereby increasing the amount of embeddable information per nucleotide from 1 bit to 2 bits. Accordingly, for a DNA sequence of length 195,005 nucleotides, the embedding capacity is increased from 195,005 bits to 390,010 bits, achieving a twofold improvement in total embedding capacity [24]. However, although only limited variations occur in the nucleotide distribution—thereby largely preserving biological consistency—the XOR-based embedding process introduces structural modifications relative to the reference DNA sequence, which moderately affects compatibility with natural sequences. In order to mitigate this structural distortion and the limited randomness introduced by the XOR operation, the integration of four-dimensional hyperchaotic systems has been proposed [25]. In the proposed method, hyperchaotic systems possessing the property Divergence = 0 generate a wide phase space and high randomness, while DNA-XOR-based DNA-oriented confusionand diffusion operations significantly improve the statistical properties of the generated sequences. Experimental results indicate that adjacent pixel correlation coefficients decrease to values close to zero in all directions, while resistance to differential attacks approaches ideal levels with NPCR = 99.60% and UACI = 33.46%. Nevertheless, the parameter sensitivity of hyperchaotic systems and the increased computational burden associated with DNA arithmetic present disadvantages, particularly for real-time applications with low latency tolerance. To alleviate this computational burden, the multilayer AES and DNA computing (MLAESDNA) method proposes a multilayer architecture that integrates the deterministic structure of AES block encryption with DNA-based transformation layers [26]. In the proposed approach, binary data are mapped to nucleotides via DNA Rule-1, after which the AES operations SubBytes, ShiftRows, MixColumns, and AddRoundKey are applied. The DNA Rule-2-based swapping stage introduces an additional biologically inspired complexity layer to the classical AES confusion–diffusion structure. However, despite efficient security metrics, the multi-key structure and multiple AES rounds supported by DNA transformations increase the computational load, particularly in biomedical and real-time applications. As an alternative to these software-oriented methods, DNA-based storage approaches that provide physical addressability have also attracted attention in the literature. The QR code-based DNA writing–reading system [27] enables information encoded in a QR code version 3 (29 × 29 pixels) structure to be converted into DNA sequences of length 93 base pairs and stored across six parallel oligonucleotides. The system provides approximately 33% redundancy and 15% error correction capability through the use of ECC level M (mol). Even when a single-bit logical error is introduced, next-generation sequencing (NGS) analyses enable the data to be recovered with 100% accuracy. Long-term stability tests demonstrate that QR patterns can be stored at room temperature for 11 months, while maintaining readability during accelerated temperature tests conducted for 2 h between −80 °C and +120 °C. Furthermore, no observable DNA damage occurs during denaturation treatments applied for up to 5 min using a 1 M NaOH solution. Nevertheless, the limited pixel capacity of the employed QR code structure, the multi-stage production process, and the potential DNA damage risk associated with deep-UV photolithography-based writing processes represent major disadvantages that restrict the scalability of the method for high-density DNA data storage systems. Another study in the traditional DNA storage literature aimed at improving coding efficiency proposes the DNA Fountain method [28]. In the proposed approach, the Luby Transform-based rateless coding structure provides high robustness against oligonucleotide loss and achieves a net information density close to the Shannon capacity. However, the intensive biochemical screening process applied to satisfy GC content and homopolymer constraints results in disadvantages such as high computational cost and reduced coding efficiency. To address these limitations, the BO-DNA approach has been proposed, incorporating a rule-based mapping mechanism and a Customized Moth–Flame (CMF) optimization algorithm based on chaotic mapping [29]. The CMF algorithm aims to maximize coding lower bounds and mitigate nonspecific hybridization errors by avoiding local optima through population diversity enhanced via tent chaotic mapping. Experimental results demonstrate that this approach reduces the need for screening by directly optimizing biological constraints and achieves significant improvements compared with previous methods in the literature. Similarly, the proposed MOPE method presents a two-stage coding architecture designed to maximize net information density [30]. Through non-payload segments designed via Modified Barnacles Mating Optimization (MBMO) and payload data encoded using the Payload Encoding (PE) algorithm, a theoretical density as high as 1.90 bits/nt is achieved. However, strict sequence constraints narrow sequence diversity and reduce primer orthogonality in large-scale oligonucleotide pools, thereby increasing the risk of cross-hybridization. Overall, the reduction from the theoretical capacity level of 2 bits/nt to the practical range of 1.57–1.90 bits/nt in DNA-based data storage systems clearly demonstrates the direct influence of biochemical and sequence-level constraints—such as GC content balance, homopolymer (base repetition) limitations, and minimum Hamming distance requirements—on system entropy. These constraints limit randomness in the sequence space and consequently suppress net information density. For this reason, studies reporting high densities should explicitly present the net user data density obtained after the application of error correction codes (ECC) and sequence constraints. Otherwise, the reported metrics remain insufficient to accurately represent the true archival capacity of the system. In conclusion, although each of the existing methods provides efficiency in certain performance metrics, simultaneously achieving high information density, biological feasibility, and scalable access requirements remains an open research problem. In this context, the development of metaheuristic and hybrid coding approaches capable of directly optimizing sequence constraints—such as GC imbalance, Hamming distance, and base repetitions—while preserving sequence diversity and expanding the search space, has emerged as an inevitable and critical research direction in the DNA-based data storage literature.

### 1.2. Metaheuristic and Intelligent Optimization Approaches in DNA Data Storage

In DNA-based data storage systems, the sequence design problem constitutes a highly complex optimization task that requires the simultaneous optimization of conflicting objectives—such as biochemical feasibility, information density, and sequence diversity—within a high-dimensional and discrete search space. Ensuring GC content balance, limiting homopolymer repetitions, and enforcing minimum Hamming distance constraints significantly narrow the feasible sequence space, thereby making it considerably difficult for classical rule-based or deterministic coding approaches to reach globally optimal solutions. This situation directly limits the scalability of the system by reducing sequence diversity, weakening primer orthogonality, and increasing the risk of cross-hybridization, particularly in large-scale DNA libraries. Sequence design problems in DNA-based data storage systems—such as biochemical feasibility, information density, and sequence diversity—are therefore treated as complex optimization problems requiring the simultaneous optimization of conflicting objectives within a high-dimensional and discrete search space. The imposition of constraints such as GC content balance, minimum Hamming distance, and prevention of secondary structure formation further restricts the feasible solution space, which in turn reduces sequence diversity and increases the risk of cross-hybridization in large-scale DNA libraries [31]. Such highly constrained DNA code design problems are generally considered in the literature as combinatorial optimization problems characterized by high computational complexity in practice. Because the search space grows exponentially with sequence length, classical deterministic and rule-based methods often fail to produce competitive lower bound values in large-scale problems [32,33]. For this reason, population-based metaheuristic and evolutionary algorithms have been widely employed in the field of DNA code design. In this context, Liu et al. proposed the BPSON algorithm, which hybridizes Particle Swarm Optimization (PSO) with the Bat Algorithm (BA) for DNA code design [34]. In the proposed approach, the multi-objective optimization problem is addressed using the Fast Non-Dominated Sorting method, allowing criteria such as continuity, hairpin structure, similarity, and H-measure to be optimized simultaneously without reducing them to a single scalar objective function. In experiments conducted on sets consisting of ten DNA codes with a length of twenty nucleotides, the BPSON algorithm produces values close to zero in terms of continuity and hairpin metrics. Furthermore, the normalized similarity metric improves to approximately 78.4 for BPSON, whereas this value remains within higher ranges for comparative NSGA-II-based methods. From a thermodynamic perspective, it is observed that GC content can be maintained at a stable level in the code sets generated by BPSON, while the melting temperature variance remains more balanced compared with competing methods. Alternatively, approaches based on the Arithmetic Optimization Algorithm (AOA) have also demonstrated numerical improvements in DNA code design. In order to generate DNA coding sets under two novel biochemical constraints—namely, pair matching and mismatch errors—the Improved Arithmetic Optimization Algorithm (IAOA) has been proposed [35]. Experiments conducted on thirteen standard benchmark functions indicate that the exploration and exploitation performance of IAOA is more efficient than that of classical AOA and similar algorithms. When DNA coding results are analyzed, it is observed that the IAOA method improves lower bound values by approximately 77.7% compared with existing algorithms, reduces melting temperature variance by between 9.7% and 84.1%, and decreases the hairpin structure ratio by between 2.1% and 80%. However, all of these results are obtained within a software simulation environment and do not include experimental validation through physical DNA synthesis [35]. Similarly, the Nonlinear Objective Learning-based Harris Hawks Optimization (NOL-HHO) method has been evaluated on 23 benchmark optimization functions in terms of convergence behavior and solution quality [36]. When applied to the DNA code design problem, measurable improvements are achieved in the lower bound values representing the capacity of the code set. Nevertheless, this study does not present detailed numerical tables regarding GC content percentages, minimum Hamming distance values, or run-length distributions, and the evaluation is largely conducted based on the mathematical optimization performance of the algorithm [36]. In the existing literature, various other metaheuristic and evolutionary algorithms—such as CLGBO [37], ROEAO [33], TMOL-TSO [38], and MFOS [39]—have also been applied to the DNA code design problem. The common characteristic of these studies is the treatment of DNA code design as a highly constrained and high-dimensional search space, where metaheuristics provide more flexible and scalable solutions compared with deterministic methods. However, in the majority of existing studies, the results remain limited to software simulations, while the stages involving physical DNA synthesis and experimental validation have not yet been sufficiently addressed.

### 1.3. Motivation and Contribution

Although DNA-based data storage systems theoretically provide a maximum information density of 2 bits per nucleotide, this theoretical upper bound cannot be attained due to biochemical and sequence-level (constraints). Requirements such as balancing GC content, eliminating homopolymer repetitions, and maintaining a minimum Hamming distance reduce the entropy of the sequence space, thereby limiting the effective information density to approximately 1.57–1.90 bits/nt. Existing coding approaches generally rely on intensive biochemical screening procedures or static sequence rules in order to satisfy these constraints. However, this situation not only increases computational costs but also suppresses sequence diversity, thereby complicating the operational sustainability of large-scale and randomly accessible DNA storage systems. Although the methods reported in the existing literature provide improvements in metrics such as net information density and error tolerance, they remain insufficient in simultaneously optimizing biological feasibility and high data density within high-dimensional and variable-length sequence spaces. In particular, within high-dimensional combinatorial search spaces, the tendency of classical metaheuristics to lose convergence stability and become trapped in local optima under constraint violations renders the need for scalable and hybrid solution mechanisms inevitable. In this context, the primary motivation of this study is to develop a flexible coding framework that defines sequence constraints not as external filtering mechanisms but as intrinsic guiding parameters of the optimization process, while maintaining stable performance across different data density regimes. In this study, a novel TC-HUR-based optimization framework is presented, which hierarchically integrates the global exploration capability of the HGS algorithm with the local refinement capacity of the RUN algorithm, with the objective of balancing the structural trade-off between biochemical constraints and net information density. The principal contributions of this study to the literature are summarized below:

- High-Dimensional and Hybrid Optimization Architecture:A novel TC-HUR architecture is proposed in which the HGS and RUN algorithms are structured in a synergistic framework. Within this architecture, the global exploration capability is enhanced through the Cauchy jump operator, while nucleotide-level constraint satisfaction is maximized through an “Adaptive Intensive Mutation” layer.
- Robustness Evaluation on High-Dimensional Complex Data Structures: In order to demonstrate the effectiveness of the proposed algorithm in challenging and high-dimensional search spaces, the analysis extends beyond the standard short sequences commonly used in the literature and validates biophysical constraint management ($GC,HP,T_{m},\Delta G$) and convergence stability on a complex dataset consisting of sequences with a length of 1853 nucleotides.
- High-Dimensional Search Space and Scalar Scalability Analysis: In order to improve the quality of sequence units that constitute the fundamental building blocks of practical DNA storage libraries, optimization is performed on a singular and complex block with a length of 1853 nucleotides. This approach provides a challenging benchmark that demonstrates the algorithm’s capacity to manage biophysical constraints ($GC,HP,T_{m},\Delta G$) and maintain convergence stability within massive search spaces (high-dimensional search space) prior to full library-scale interactions.
- Error-Aware Robustness Analysis: In order to evaluate the laboratory feasibility of the optimized sequences, comprehensive error simulations are conducted under different noise regimes using the DNAStoralator platform, and nucleotide-level error patterns are analytically examined.
- SOTA-Based Comparative Performance Evaluation: The TC-HUR architecture is compared with contemporary state-of-the-art (SOTA) algorithms such as IAOA, GWO, and PSO in terms of critical metrics including convergence rate, sequence diversity, and thermodynamic stability, thereby demonstrating its methodological efficiency in high-dimensional optimization problems.

## 2. Results

### 2.1. Experiment Setup

All numerical simulations conducted in this study were performed on a computational platform equipped with an Intel Core i5-10300H processor (2.50 GHz) and 16 GB of RAM, running a 64-bit Windows 11 operating system using the Python 3.12 programming language. To minimize the variance effects arising from the stochastic nature of the algorithms, each experiment was independently repeated 30 times, and performance evaluations were carried out based on the mean and standard deviation values. During the optimization process, the initial population size and the maximum number of iterations were fixed as N=50 and $T_{max}=100$, respectively, for all algorithms. In order to objectively evaluate the performance of the proposed TC-HUR framework and the comparison algorithms including PSO, GWO, HGS, and RUN, as well as the recently prominent State-of-the-Art (SOTA) Improved Arithmetic Optimization Algorithm (IAOA) [35], algorithm-specific control parameters commonly adopted in the literature were employed for each method. In the Particle Swarm Optimization (PSO) algorithm, the parameters $c_{1},c_{2}=2.05$ and w=0.4 ensure the cognitive–social balance, whereas in the Grey Wolf Optimization (GWO) model, the linear decrease of the parameter *a* regulates the transition from exploration to exploitation. The RUN algorithm utilizes the Enhanced Solution Quality (ESQ) mechanism, inspired by numerical integration techniques, to improve solution quality. The primary parameters of this mechanism, a=20 (randomness control) and f=0.1 (step-size scaling), regulate exploitation sensitivity and stable convergence in high-dimensional search spaces. In the Hunger Games Search (HGS) model, the parameters PUP=0.08 and LH = 10,000 preserve the hunger-driven search dynamics. For the IAOA algorithm, dynamic operator selection is performed through the parameters α=5 and μ=0.5. Within the proposed TC-HUR hybrid architecture, the initial Cauchy jump intensity $\beta_{start}=0.015$ and the exploitation-phase coefficient $f_{refine}=0.008$ combine the mathematical exploitation capability of the RUN algorithm with fine-grained improvements at the nucleotide level. In addition, the adaptive mutation rate increasing from 2% to 5% (0.02→0.05) optimizes persistent constraint violations—such as homopolymer formation and reverse-complement (RC) similarity—in complex sequences with high precision. Detailed parameters and configurations of the related algorithms are presented in Table 1.

The DNAStoralator simulator [40], used to evaluate the robustness of the designed nucleotide sequences under biological error mechanisms, emulates the PCR amplification and sequencing processes encountered in laboratory environments. In DNA-based data storage systems, the performance of sequences is determined not only by their static biophysical feasibility but also by their resilience against error profiles arising during synthesis, amplification, and sequencing stages. For this reason, robustness analysis is performed under multiple channel configurations representing different error spectra rather than a single abstract error model. For this purpose, the simulation environment parameterizes ONT MinION (an Oxford Nanopore-based long-read sequencing platform) as the sequencing technology and Integrated DNA Technologies (IDT synthesis profile) as the synthesis technology. The ONT MinION configuration represents the nanopore error model characterized in the literature by high insertion–deletion (indel) rates and particularly long deletion events. This configuration enables the evaluation of the effects of frame-shift errors and cumulative indel impacts observed in long-read-based systems on the designed sequences. In contrast, the IDT synthesis profile is modeled to include moderate substitution rates, short single-base deletions, and error tendencies observed particularly at GC terminal regions and within homopolymeric segments. Although this profile does not contain indel events as intensively as nanopore-based sequencing, it remains critically important in reflecting the accumulation of errors related to GC content and homopolymer length. Since GC balance and homopolymer suppression constitute two fundamental biophysical axes of the optimization process in this study, this profile provides stress conditions that are directly aligned with the design objectives of the algorithms. The stress testing procedure refers to evaluating the capacity of the designed DNA sequences to preserve data integrity under high-error-rate conditions by simulating sequencing errors such as substitution, insertion, and deletion events. When these two complementary configurations are considered together, a broad spectrum of the primary error mechanisms encountered in DNA data storage systems is represented, including the high-indel-dominant long-read channel and the GC/homopolymer-sensitive synthesis channel. The optimized sequences are degraded under low, medium, and high noise levels and comparatively analyzed in terms of normalized edit distance (NED), substitution, insertion, and deletion rates. In this way, the robustness of biophysically optimized sequences is evaluated under parameters that represent synthesis- and sequencing-induced error spectra expected in laboratory environments, thereby enabling the analysis of their capacity to preserve structural integrity under realistic process conditions. This approach demonstrates that the results are not limited to a specific abstract simulation environment but are instead evaluated under multiple channel models representing different realistic error profiles, thereby grounding the robustness claims within the framework of platform-dependent error spectra. The parametric distribution ranges corresponding to the low, medium, and high error regimes defined for the experimental evaluation are presented in Table 2. Through this comprehensive experimental design, the reference sequences generated by the algorithms are subjected to analysis based on normalized edit distance, nucleotide context–dependent error rates, and biological stability metrics. In particular, the tendency of long homopolymer repeats to induce errors during PCR processes is considered a key performance indicator for evaluating the design quality of the algorithms.

### 2.2. Experiment Results

**Figure 1:**
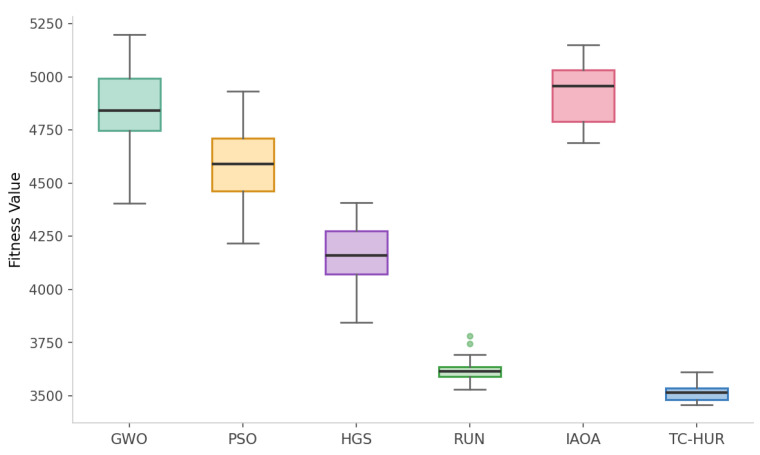
The distribution of fitness values obtained by six metaheuristic algorithms over 30 independent runs is illustrated. The box plots present the median, interquartile range (IQR), and minimum–maximum values for the GWO, PSO, HGS, RUN, IAOA, and TC-HUR algorithms. Outliers are indicated as individual points. The Friedman non-parametric test confirms that statistically significant differences exist among the algorithms ($\chi^{2}=137.03$, p<0.001).

**Figure 2:**
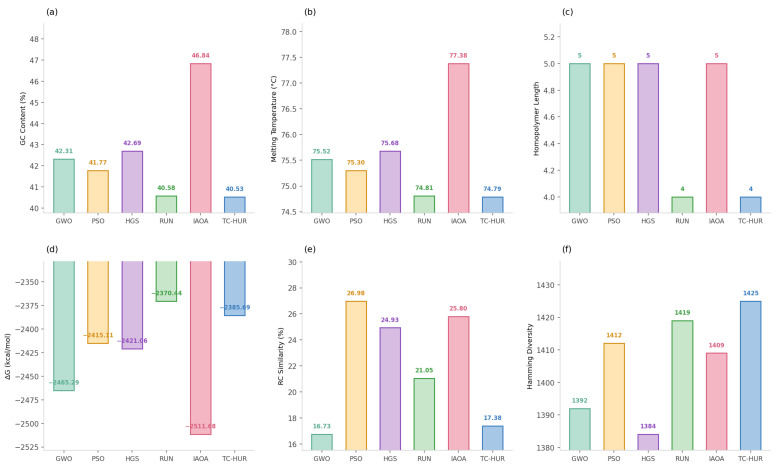
Biophysical quality metrics of the best DNA sequences generated by the GWO, PSO, HGS, RUN, IAOA, and TC-HUR algorithms. The panels respectively present (**a**) GC content (%), (**b**) melting temperature ($T_{m}$, °C), (**c**) maximum homopolymer length (HP), (**d**) thermodynamic free energy (ΔG, kcal mol^−1^), (**e**) reverse-complement similarity (RC, %), and (**f**) Hamming diversity. Each panel is presented as a bar chart, and the numerical labels above the bars indicate the exact value of the corresponding metric.

**Figure 3:**
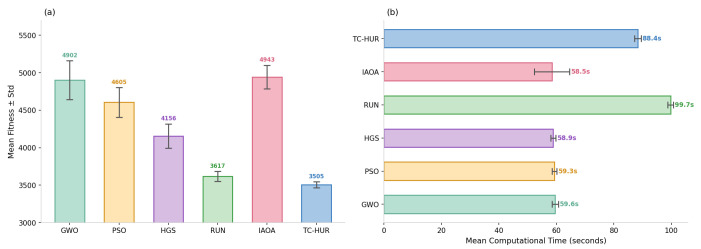
Mean fitness values and average computational times of the six metaheuristic algorithms. Panel (**a**) presents the mean fitness values of the GWO, PSO, HGS, RUN, IAOA, and TC-HUR algorithms together with ±1 standard deviation error bars. Panel (**b**) illustrates the average computational time per run (seconds) in the form of a horizontal bar chart with ±1 standard deviation error bars.

**Figure 4:**
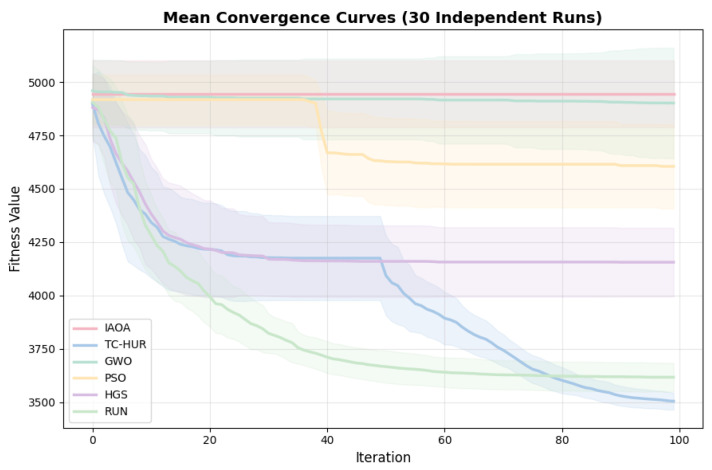
Average convergence curves of TC-HUR and the SOTA algorithms. The mean fitness values obtained from 30 independent runs on the 1853 nt dataset are presented, while the shaded regions represent the ±SD intervals reflecting the stochastic stability of the algorithms (n=30).

In this section, the performance analysis of the proposed TC-HUR algorithm and the metaheuristic methods included in the comparison set is conducted for the design of a DNA sequence with a length of 1853 nucleotides. The results in terms of both optimization quality and biophysical constraint efficiency are presented in Figure 1, Figure 2, Figure 3 and Figure 4. The detailed numerical results corresponding to the evaluated metrics are provided in Table 3. Across 30 independent runs, the TC-HUR algorithm achieves the most optimal result among all compared algorithms with an average fitness value of 3504.99 ± 39.24. All other algorithms, including GWO (4901.77 ± 259.10), IAOA (4943.48 ± 157.02), PSO (4604.82 ± 197.52), HGS (4156.03 ± 161.17), and RUN (3617.21 ± 67.30), produce significantly higher fitness values. TC-HUR also exhibits the smallest standard deviation among all algorithms (σ=39.24), demonstrating superior convergence stability. The Friedman non-parametric test confirms that the differences among the algorithms are statistically significant ($\chi^{2}=137.03$, p<0.0001). Subsequently, pairwise comparisons conducted using the Wilcoxon signed-rank test reveal that TC-HUR significantly differs from all competing methods (p<0.05). The only pair for which no significant difference is observed is the IAOA–GWO pair, confirming that these two algorithms exhibit similar performance levels (p=0.655). Homopolymer and reverse-complement (RC) analyses directly reflect the primary sources of errors in DNA synthesis and sequencing processes. The IAOA, GWO, PSO, and HGS algorithms all produce homopolymer sequences of length 5, whereas RUN reduces this value to 4. TC-HUR, together with RUN, achieves HP=4, thereby sharing the best performance among the evaluated algorithms. In terms of RC similarity, TC-HUR achieves the lowest value among all methods with 17.38%, indicating that the risk of secondary structure formation is effectively suppressed. While PSO (26.98%) and HGS (24.93%) produce the highest RC similarity values, RUN (21.05%) and GWO (16.73%) demonstrate moderate performance levels. The efficiency of TC-HUR in suppressing RC similarity thermodynamically minimizes the risk of hairpin and self-dimer formation within the sequence. The obtained RC similarity value of 17.38% demonstrates the capability of the Cauchy jump operator to disrupt successive complementary nucleotide motifs during the global exploration phase, thereby reducing undesirable hybridization affinity. This behavior indicates that the heavy-tailed Cauchy distribution, unlike Gaussian-based approaches, can destabilize local sequence regions prone to secondary structure formation within a single iteration, thereby providing a more reliable DNA data storage layer. Hamming distance is considered a fundamental metric for evaluating the error tolerance and distinguishability of DNA sequences. TC-HUR achieves the highest sequence diversity among all algorithms with a Hamming value of 1425, outperforming IAOA (1409), RUN (1419), GWO (1392), PSO (1412), and HGS (1384). This result indicates that TC-HUR maintains sequence heterogeneity while strictly enforcing biophysical constraints. All algorithms generate GC contents within the biologically acceptable range of 40–60%. TC-HUR (40.53%) and RUN (40.58%) produce balanced compositions closer to the lower boundary of this range. In contrast, IAOA (46.84%), PSO (41.77%), and GWO (42.31%) exhibit higher GC densities, which may increase the risk of secondary structure formation. IAOA produces the sequence with the lowest free energy (−2511.68 kcal/mol), which indicates excessively strong binding and potential PCR denaturation difficulties. Similarly, GWO (−2465.29 kcal/mol) and PSO (−2415.11 kcal/mol) also generate overly stable sequences. In contrast, TC-HUR (−2385.69 kcal/mol), together with RUN (−2370.44 kcal/mol) and HGS (−2421.06 kcal/mol), demonstrates a more balanced energy profile that preserves sufficient thermal stability while limiting excessive binding effects. In terms of melting temperature, all methods produce values above the target reference range of 55–65 °C. However, due to the length of the sequences considered in this study and the simultaneous optimization of multiple biochemical constraints, these values are treated as reference targets within the optimization process. Nevertheless, the $T_{m}$ value obtained by TC-HUR (74.79 °C) is lower than those of IAOA (77.38 °C) and PSO (75.30 °C), indicating a more controlled thermal dissociation behavior. When considered together with the ΔG results, TC-HUR is observed to produce the thermodynamically most balanced sequence among all evaluated algorithms. In terms of computational efficiency, TC-HUR operates faster than the RUN algorithm with an average runtime of 88.36 s per run compared to 99.72 s. However, IAOA (58.47 s), GWO (59.59 s), PSO (59.32 s), and HGS (58.91 s) offer lower computational times of approximately 59–60 s. This additional computational cost arises from the two-phase hybrid architecture of TC-HUR, which combines HGS-based exploration with RUN-based refinement stages. Nevertheless, considering the biophysical quality improvements provided by TC-HUR, particularly the superior RC suppression (17.38%), the highest Hamming diversity (1425), and the lowest fitness variance (σ=39.24), this additional computational cost represents an acceptable trade-off for obtaining biologically reliable and synthesizable DNA sequences.

The average convergence curves obtained from 30 independent runs of the compared optimization algorithms are presented in Figure 4. The TC-HUR algorithm demonstrates the best convergence performance with an average fitness value of 3497.27, producing approximately 2.5% better results than the RUN algorithm and 16.7% better results than the HGS algorithm. In addition, the standard deviation value of TC-HUR (31.60) indicates a more stable and repeatable optimization process compared to PSO (204.83) and HGS (235.82).

#### 2.2.1. Error-Aware Comparative Performance Under Noisy DNA Storage Channels

**Figure 5:**
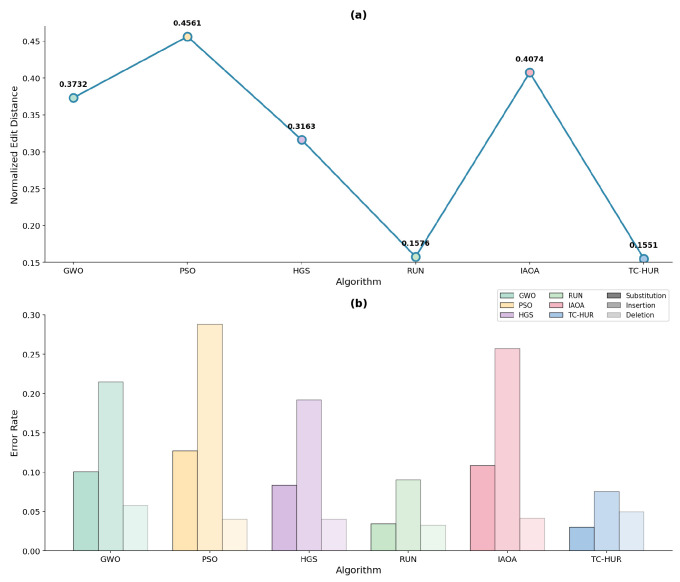
Algorithmic performance comparison under the low-noise regime. (**a**) Distribution of normalized edit distance (NED) values ($NED_{TC\text{-}HUR}=0.1551$); (**b**) The composition of error types under the low-noise regime, where the grayscale gradient from dark to light represents substitution, insertion, and deletion rates, respectively.

**Figure 6:**
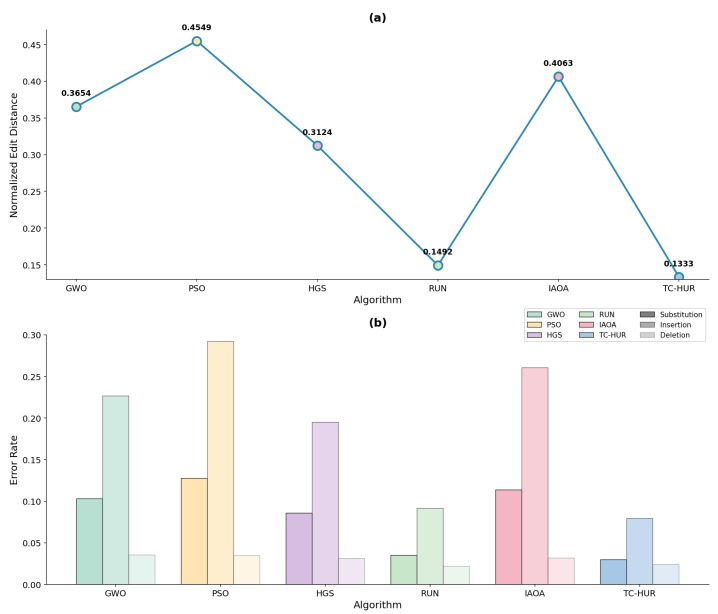
Error component analysis under the medium-noise regime ($NED_{TC\text{-}HUR}=0.1333$). (**a**) Comparative NED results across the evaluated algorithms; (**b**) The composition of error types under the medium-noise regime, where the grayscale gradient from dark to light represents substitution, insertion, and deletion rates, respectively.

**Figure 7:**
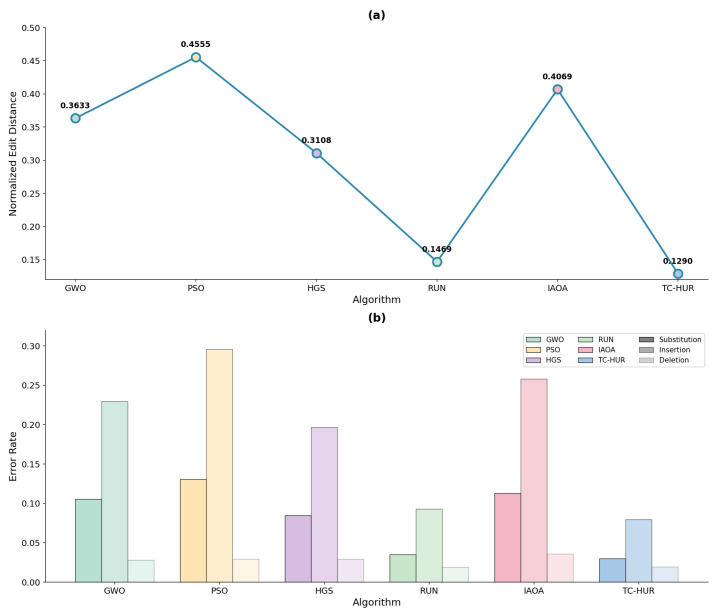
Maximum error resilience analysis under the high-noise regime ($NED_{TC\text{-}HUR}=0.1290$). (**a**) NED performance of the evaluated algorithms; (**b**) The composition of error types under the high-noise regime, where the grayscale gradient from dark to light represents substitution, insertion, and deletion rates, respectively.

The biophysical optimization results and Table 3 presented in the previous section demonstrate the ability of the algorithms to generate synthesizable, thermodynamically stable DNA sequences that satisfy biological constraints. The ranking and numerical values associated with the NED results for all error levels are provided in Table 4. The algorithmic performance results corresponding to the low, medium, and high noise regimes are illustrated in Figure 5, Figure 6, and Figure 7, respectively. According to these results, the proposed TC-HUR algorithm consistently achieves superior performance across all error levels. At the low error level, TC-HUR ranks first with a NED value of 0.1551, while its closest competitor, RUN, exhibits a slightly less efficient performance with a value of 0.1576, corresponding to approximately 1.6% lower efficiency. In contrast, pure swarm intelligence-based methods such as HGS (0.3163) and GWO (0.3732) produce error levels more than twice that of TC-HUR. At the medium error level, the NED value of TC-HUR decreases to 0.1333, whereas RUN remains at 0.1492. This indicates that TC-HUR achieves an edit distance efficiency approximately 10.6% higher than RUN. Under the same conditions, the PSO algorithm remains at a substantially high value of 0.4549, revealing its pronounced vulnerability to channel noise. Under the high error regime, the performance gap becomes even more pronounced. The NED value of TC-HUR (0.1290) is approximately 12.2% more efficient than the RUN result (0.1469). In comparison, the PSO value of 0.4555 is about 3.5 times less efficient than TC-HUR. These findings indicate that TC-HUR maintains stability not only under low-noise conditions but also under realistic high-error scenarios. When the sub-error components are examined, TC-HUR maintains substitution rates at approximately the 0.03 level across all noise regimes. For instance, under the high error level, TC-HUR produces a substitution rate of 0.0300, while RUN, HGS, and PSO exhibit values of 0.0351, 0.0847, and 0.1305, respectively. This behavior indicates that TC-HUR systematically suppresses nucleotide-level mismatch errors within the sequence structure. A similar trend is observed for insertion rates. Under high error conditions, TC-HUR produces an insertion rate of 0.0794, compared with 0.0928 for RUN, 0.1965 for HGS, and 0.2955 for PSO. The approximately 14% lower insertion error produced by TC-HUR relative to RUN indicates that the addition of the HGS phase to the strong local search capability of RUN enables better control over homopolymer formation and sequence density patterns. This tendency arises not only from the global search capability of TC-HUR but also from the cooperative interaction between the RUN-based local refinement mechanism and the HGS-based population diversity mechanism. While the RUN mechanism suppresses local motif errors within sequences, the HGS phase simultaneously optimizes global biological constraints such as homopolymer formation and GC imbalance. The combined operation of these two mechanisms enables TC-HUR to continue reducing the NED value even as the error level increases. When deletion rates are examined, all algorithms produce relatively low and similar values. Under high error conditions, TC-HUR produces a deletion rate of 0.0195, while RUN, HGS, and PSO exhibit values of 0.0190, 0.0295, and 0.0294, respectively. These results indicate that deletion errors generally exhibit limited variation and do not produce a strong distinction among algorithms. Nevertheless, TC-HUR maintains sequence stability by generating lower deletion rates compared with HGS- and PSO-based methods. This outcome demonstrates that TC-HUR not only suppresses insertion and substitution errors effectively but also maintains deletion errors at consistently low levels.

#### 2.2.2. Homopolymer Sensitivity Analysis Under Noisy DNA Storage Channels

The ability of the evaluated algorithms to control homopolymer (HP) formation and the associated error patterns under channel noise is presented in Table 5. The effectiveness of each algorithm is characterized not only in terms of the static maximum HP length but also through a holistic assessment involving HP density and homopolymer-induced insertion–deletion (indel) error rates. Under the low and medium noise regimes, TC-HUR maintains the average maximum homopolymer length (Avg Max HP) at 5.68 bp and 5.08 bp, respectively, outperforming its closest state-of-the-art competitor, IAOA, which produces 6.29 bp and 5.94 bp under the same conditions. Furthermore, under the high-noise regime, TC-HUR achieves an Avg Max HP value of 4.89 bp, surpassing RUN (4.96 bp), HGS (5.79 bp), IAOA (6.18 bp), and GWO (6.35 bp). These results demonstrate that the hybrid architecture effectively suppresses nucleotide repetitions even under extreme mutational load. Overall, the data confirm that TC-HUR reduces homopolymer length by approximately 20% compared with IAOA and by about 23% compared with GWO and PSO. When examined in terms of homopolymer density (Avg HP Count), TC-HUR consistently produces the lowest number of HP regions across all noise levels. Under the high-noise regime, the obtained value of 80.19 is approximately 22% lower than that of IAOA (102.64) and 31% lower than that of GWO (116.42). The ability of the proposed method to minimize not only HP length but also the density of HP motifs within the sequence systematically reduces the risk of polymerase slippage. This success in biochemical constraint management directly translates into improved robustness against channel noise. Homopolymer length and density are known to be primary triggers of insertion and deletion errors in DNA data storage systems. Although the differences among algorithms narrow when considering HP-induced indel percentages, TC-HUR still exhibits the lowest error margin under high-noise conditions with a rate of 13.8%. Compared with the state-of-the-art approaches IAOA (13.9%) and RUN (14.7%), as well as HGS (14.4%), GWO (14.9%), and PSO (14.0%), TC-HUR demonstrates a more resilient error profile. The consistent performance of the proposed method across all noise regimes can be attributed to the targeted constraint refinement capability of the Adaptive Intensive Mutation layer at the nucleotide level. While other algorithms remain more susceptible to homopolymer-related constraint violations in high-dimensional sequence spaces, the nucleotide-level refinement mechanism in TC-HUR preserves sequence integrity and systematically suppresses indel errors even under severe channel noise conditions. Consequently, the low homopolymer profile observed for TC-HUR in Table 5 is directly consistent with the reduced insertion and deletion rates reported in Table 4. In particular, the ability of TC-HUR to simultaneously produce the lowest Max HP value and the lowest overall indel rates under high-error conditions confirms that effective biophysical constraint control can directly mitigate channel-induced noise effects.

**Figure 8:**
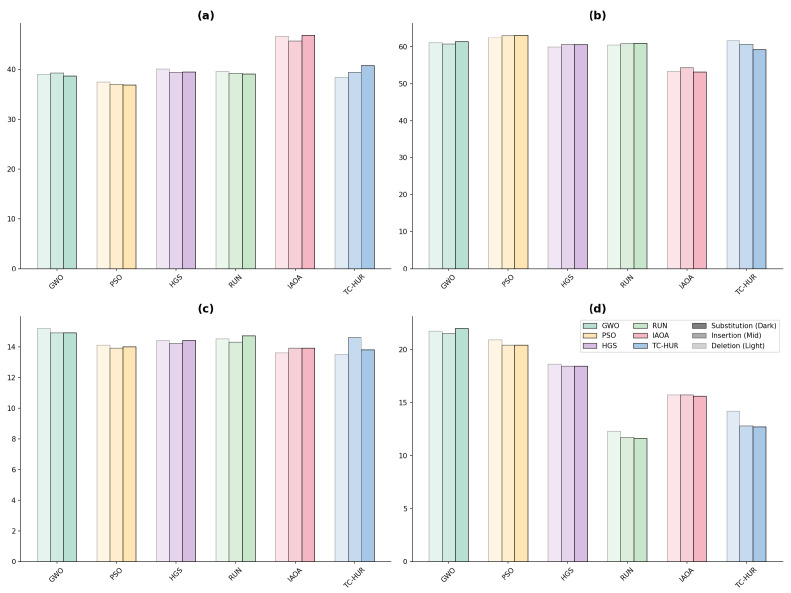
Context-dependent sequencing error analysis under three error regimes (Low, Mid, High) for six metaheuristic algorithms on a 1853 nt DNA storage sequence. Values are normalized to the total number of substitutions or indels per algorithm. (**a**) GC→AT and (**b**) AT→GC substitution rates represent the proportion of each transition type among all substitutions. (**c**) Homopolymer-associated indel rate and (**d**) homopolymer-associated substitution rate denote the fraction of errors occurring within homopolymer regions. Error regimes are indicated by bar opacity (light: Low, medium: Mid, dark: High).

Error characteristics in DNA data storage systems are directly associated with nucleotide composition and sequential context. Within this scope, the conducted analysis holistically evaluates nucleotide-based error patterns of the algorithms, base transition tendencies, and homopolymer-induced error sensitivities. Numerical data are presented in Table 6, while visual comparisons are illustrated in Figure 8. When the correlation between biochemical constraint management and channel errors is examined, TC-HUR exhibits a balanced error profile even under a high-noise regime. The fact that TC-HUR possesses GC→AT 40.8% and AT→GC 59.2% values at a high error level confirms that the ideal GC balance within the 40–60% range is preserved even under noise. In contrast, different findings emerge when compared with other algorithms. The 36.9–63.1% distribution of PSO indicates a critical deviation in GC content, leading to imbalance in nucleotide composition. Similarly, GWO demonstrates a comparable deviation tendency with ratios of 38.7–61.3%. The 46.9–53.1% ratio of IAOA aggressively increases GC density. This situation reflects that, although the IAOA architecture prioritizes thermodynamic stability, it remains insufficient in controlling context-dependent errors. Although the HGS algorithm presents a profile close to TC-HUR with 39.5–60.5%, it exhibits higher NED values in homopolymer-based errors. In terms of homopolymer-based error components (HP-Sub), the RUN algorithm achieves the lowest values with 11.6%. However, the close performance of TC-HUR with 12.7% is balanced by its efficiency in the HP-Indel rate. The fact that the HP-Indel rate of TC-HUR is lower than RUN (13.8% vs. 14.7%) reveals that although RUN is successful in suppressing base substitutions, it exhibits a more fragile structure against insertion–deletion errors. Consequently, TC-HUR provides an optimized trade-off point over context-dependent errors. Rather than excessively optimizing a specific error type while leaving others uncontrolled, the algorithm simultaneously limits homopolymer-induced indel generation while preserving GC balance. At a high error level, both the preservation of the ideal nucleotide distribution and the suppression of indel rates constitute the biophysical basis of the methodological efficiency that TC-HUR provides against its NED competitors.

## 3. Discussion

The experimental findings obtained in this study demonstrate that the proposed TC-HUR algorithm not only provides a theoretical optimization success for DNA data storage systems but also produces sequences that are biophysically synthesizable and capable of preserving their structural integrity under channel noise. The efficiency offered by the methodology is characterized along three primary axes: convergence stability, precise constraint satisfaction, and thermodynamic balance. First, the average fitness value of 3497.27 obtained by TC-HUR on the high-dimensional poetry dataset consisting of 1853 nucleotides, together with a low standard deviation of 31.60, reflects the capability of the hybrid architecture to avoid local optimum traps on complex constraint surfaces. While classical metaheuristic approaches (IAOA, GWO, PSO) are observed to either fail to suppress constraint violations or fail to preserve sequential entropy within this dimensional space, TC-HUR harmonizes HGS-based global exploration with RUN-based local refinement, thereby simultaneously managing both local motif errors and global sequence imbalance. From a thermodynamic perspective, the melting temperature values presented in Table 3 ($T_{m}\approx 74.88$ °C) and reverse complement similarity (RC≈19.10%) remain above the nominal target thresholds defined in the objective function (55–65 °C and 15%). This situation arises as an optimization outcome originating from the nature of the quadratic penalty functions employed, which are managed as soft constraints within a high-dimensional search space. Under this condition, the algorithm satisfies the homopolymer limits (Max HP ≤3), which are critically important for synthesizability, while converging to a near-optimal trade-off point that preserves the overall system stability and structural robustness in terms of the $T_{m}$ and RC parameters. Sequences with excessive stability (low ΔG) produced by methods such as IAOA and PSO may lead to kinetic traps during PCR denaturation, whereas the balanced energy profile provided by TC-HUR limits secondary structure formation and enhances operational sustainability. The reflection of these biophysical advantages on channel performance is clearly observed in error-aware experiments conducted under the DNAStoralator simulation environment. The fact that TC-HUR produces the lowest NED values (0.1551–0.1290) across all error regimes indicates that the sequences preserve their structural integrity even under realistic channel noise. In particular, the maximum homopolymer length value of 4.89 bp obtained in the homopolymer sensitivity analysis minimizes the enzymatic slippage tendency of the polymerase enzyme, thereby suppressing insertion–deletion (indel) error rates 14% more effectively compared with the RUN algorithm. In terms of computational cost, although TC-HUR (89.09 s) exceeds the baseline runtime of approximately 60 s offered by classical algorithms, its ability to reduce constraint violations within biological limits (HP ≤3) compensates for this cost by making it the most efficient method. Considering the costs associated with DNA synthesis and sequencing, this additional computational overhead at the design stage constitutes a negligible trade-off compared with the experimental repetition costs that may arise from unsynthesizable or incorrectly encoded sequences. Consequently, TC-HUR provides a reliable design framework for future scalable DNA storage architectures by balancing biophysical constraints and channel error mechanisms from a holistic perspective.

### 3.1. Comparative Analysis of the TC-HUR Algorithm with Specific Limitations in the Literature

In this section, the efficiency of the proposed TC-HUR algorithm over widely used DNA coding strategies in the literature is analyzed through the specific mechanistic weaknesses of existing algorithms and the capacity of the proposed method to improve these limitations. From the perspective of search dynamics and convergence stability, algorithms prominent in the literature such as IAOA and GWO exhibit limited exploration capability in high-dimensional (1853 nt) and heavily constrained solution spaces. The tendency of IAOA to lose exploration pressure early in complex domains and stagnate in suboptimal regions, together with the static transition structure introduced by the linear parameter reduction of GWO, leads to inefficiency in large-scale DNA sequence design. TC-HUR becomes more efficient under these structural limitations through its tri-phase dynamic mechanism and heavy-tailed perturbations based on the Cauchy distribution (Cauchy jump). Following the global exploration phase, the algorithm transitions into a RUN-based intensive local search stage, enabling the population to escape local optimum traps. When Table 3 is examined, the average fitness value of TC-HUR (3497.27), compared with IAOA (4895.97) and GWO (4936.03), confirms that the proposed method achieves approximately 28–29% more stable convergence while systematically suppressing constraint violations. In terms of precise constraint management and homopolymer control, studies such as BO-DNA and BPSON generally perform homopolymer control using a passive strategy based on global penalty functions. This approach remains insufficient in refining homopolymer blocks at the nucleotide level and may lead to erroneous repetitions with lengths of 4–5 bp. TC-HUR improves this limitation through the Adaptive Intensive Mutation layer, which enables intervention at the nucleotide level. During the iterative process, this layer, whose intensity increases from 2% to 5%, performs refinements with surgical precision by targeting critical violations such as homopolymers and reverse-complement similarity. Experimental results demonstrate that TC-HUR is the only method capable of stabilizing homopolymer length at the biologically ideal threshold of 3 bp. Furthermore, maintaining the maximum homopolymer length (Max HP) at the level of 4.89 bp even under high-error regimes proves that TC-HUR exhibits 6% more effective constraint management compared with the RUN algorithm. When examined from the perspective of thermodynamic balance and operational robustness, extremely low free energy (ΔG) values produced by algorithms such as PSO and IAOA (respectively −2423.28 and −2496.43 kcal/mol) indicate excessive molecular stability, which may lead to kinetic traps during PCR amplification and difficulties in thermal denaturation (melting). TC-HUR synchronizes global exploration and local refinement processes, stabilizing the ΔG value at −2384.87 kcal/mol, which lies within the biologically ideal equilibrium range. In channel noise analyses, although the RUN algorithm demonstrates success in suppressing local motif errors, the global and local search synchronization provided by TC-HUR yields a 12.2% more successful NED (0.1290) performance under high error levels. This finding reveals that TC-HUR is not merely a static design tool but also a coding paradigm resistant to channel errors.

### 3.2. Limitations

The experimental findings obtained in this study demonstrate that the proposed TC-HUR algorithm provides statistically significant efficiencies in terms of biophysical suitability and channel robustness in DNA sequence design. However, several fundamental limitations remain that define the practical boundaries of the methodology and highlight open research directions that should be addressed in future studies. First, the experimental processes are conducted using a fixed length of 1853 nucleotides representing the input text. Due to the high sensitivity of metaheuristic methods to the dimensionality of the search space, the convergence behavior and the capacity to escape local optima for shorter sequences (200–500 nt) or extremely long sequences (>5000 nt) have not yet been systematically analyzed. The channel model employed in this study is based on a stochastic noise simulation and does not fully capture the technology-specific base-dependent error profiles of commercial sequencing platforms such as Illumina, Nanopore, or PacBio. In addition, the fact that the findings remain limited to software-based simulations and have not been experimentally validated in a wet-laboratory (wet-lab) environment constitutes a limitation regarding the practical applicability of the method. While the current evaluation framework focuses on empirical metrics such as ΔG and $T_{m}$, real DNA behavior is determined not only by free energy minimization but also by secondary structure kinetics and complex hairpin interactions. Therefore, in future work it will be necessary to integrate minimum free energy (MFE) structures and cross-hybridization scores into the fitness function of TC-HUR. Moreover, parameter optimization and data diversity factors may directly influence the performance of the algorithm. Since these factors have not been systematically evaluated across different scenarios, the performance of the method may remain dependent on specific experimental conditions. Finally, this study performs the optimization of a single reference sequence, whereas DNA storage libraries require the simultaneous design of millions of orthogonal sequences. Consequently, extending the TC-HUR architecture into a multi-objective framework that directly minimizes inter-sequence cross-similarity (cross-talk) is critically important for improving the archival capacity and data integrity of large-scale DNA storage systems.

## 4. Materials and Methods

### 4.1. Problem Formulation and Objective Functions

The design of nucleotide sequences in DNA-based data storage systems fundamentally differs from classical numerical optimization problems. While data integrity in digital environments is typically defined through abstract error models, error formation in DNA-based systems is directly associated with molecular biology, biophysical interactions, and enzyme kinetics. Consequently, the sequence design process is treated as a constrained and high-dimensional optimization problem that requires the simultaneous balancing of multiple biophysical requirements. In this study, the problem is modeled as a minimization problem based on quadratic penalty functions, in which multiple biological objectives and constraints are unified under a single scalar fitness function. The overall fitness function is presented in Equation (1).

(1) $$Fitness=\sum_{i=1}^{3}w_{obj,i}\cdot f_{obj,i}+\sum_{j=1}^{3}w_{cons,j}\cdot C_{cons,j}$$

Here, $w_{obj,i}$ and $w_{cons,j}$ represent the weighting coefficients assigned to each objective and constraint term according to their respective levels of biophysical importance. In particular, due to their critical influence on DNA synthesizability, terms related to homopolymer length and melting temperature are prioritized with higher weights during the optimization process. The structural integrity and experimental success rate of DNA sequences primarily depend on base composition. Guanine–Cytosine (GC) base pairs form three hydrogen bonds, which provide stronger binding energy compared with Adenine–Thymine (AT) pairs, thereby directly influencing the thermal stability of the DNA double helix. For this reason, deviations of the GC ratio from the ideal value of 50% are modeled as a quadratic penalty function as expressed in Equation (2).

(2) $$f_{GC}={\left(\frac{G+C}{L}\cdot 100-50\right)}^{2}$$

Here, G+C denotes the total number of guanine and cytosine bases in the sequence, while *L* represents the total length of the sequence. In addition to nucleotide composition, homopolymer regions—arising from polymerase slippage during synthesis and sequencing processes and constituting a primary source of insertion–deletion (indel) errors—play a critical role in the design process. Considering the cumulative nature of error propagation, and in contrast to the single homopolymer penalties commonly used in the literature, all homopolymer blocks longer than three bases are minimized through a cumulative penalty mechanism as defined in Equation (3).

(3) $$f_{HP}=\sum_{k=1}^{n}{\left(h_{k}-3\right)}^{2},\;\forall \;h_{k}>3$$

Here, $h_{k}$ represents the length of the *k*-th homopolymer block within the sequence. The thermodynamic behavior of a DNA sequence is characterized by the Gibbs free energy (ΔG), which represents the stacking interactions between successive base pairs. In order to eliminate sequence-length-dependent bias, the normalized energy deviation is expressed in Equation (4).

(4) $$f_{\Delta G}=\left|\frac{\sum \Delta G_{dinucleotide}}{L}-\Delta G_{target}\right|\cdot 100$$

Here, $\Delta G_{dinucleotide}$ denotes the stacking energy of each dinucleotide step, while $\Delta G_{target}$ represents the biologically ideal value of −1.45 kcal/mol. The melting temperature ($T_{m}$), which represents thermal stability, is calculated using a salt-corrected empirical model in order to prevent experimental bias that may occur during PCR amplification, as shown in Equation (5).

(5) $$T_{m}=64.9+41\cdot \frac{{\left(G+C\right)}_{perc}-16.4}{100}$$

Here, ${\left(G+C\right)}_{perc}$ represents the percentage of guanine and cytosine bases within the sequence. The constraint violation term applied when the calculated values fall outside the defined biological target range is expressed in Equation (6).

(6) $$C_{Tm}={\left(T_{m}-T_{target}\right)}^{2},\;\mathrm{if}\;T_{m}\notin \left[55,65\right]$$

Here, $T_{target}$ represents the distance to either the lower or upper bound of the target interval. In order to preserve data security and structural stability, the minimum Hamming distance between sequences and the reverse-complement similarity that may trigger hairpin formation are defined as additional constraints. The violation of the minimum Hamming distance with respect to the existing sequence archive $A_{i}$ is penalized as defined in Equation (7).

(7) $$C_{Ham}={\left(d_{min}-\min\left(d\left(S,A_{i}\right)\right)\right)}^{2},\;\mathrm{if}\;\min\left(d\right)<d_{min}$$

Here, $d(S,A_{i})$ denotes the distance between the candidate sequence *S* and the archive sequences, while $d_{min}$ represents the biologically acceptable minimum distance threshold. Similarly, the penalty term applied when the reverse-complement similarity ratio exceeds the threshold of 15% is presented in Equation (8).

(8) $$C_{RC}=\left(similarity\_ratio-0.15\right)\cdot 100,\;\mathrm{if}\;similarity\_ratio>0.15$$

Here, similarity_ratio denotes the similarity ratio between the sequence and its own reverse complement. All biophysical constraints formulated in this study are integrated into the optimization algorithm through “quadratic penalty functions,” which are widely employed in high-dimensional optimization problems. Rather than enforcing rigid boundaries (hard constraints), this approach defines constraints as “soft constraints” that guide the system toward desired values by incorporating high weighting coefficients ($w_{cons}$) into the fitness function. However, within large search spaces, satisfying multiple conflicting biophysical objectives simultaneously with zero deviation mathematically requires an inherent trade-off.

### 4.2. Proposed Tri-Phase Cauchy-Assisted HGS and Unified RUN (TC-HUR)

In this study, a hybrid optimization architecture termed Tri-phase Cauchy-assisted HGS and Unified RUN (TC-HUR) is proposed to effectively address the high-dimensional and highly constrained optimization problems encountered in DNA data storage systems. The TC-HUR framework integrates three complementary components within a unified hierarchical structure: the high global exploration capability of the HGS algorithm, the mathematically driven exploitation ability of the RUN-based local refinement stage, and an Adaptive Intensive Mutation layer designed to resolve persistent constraint violations at the nucleotide level. The evolutionary process of the algorithm consists of three principal phases that provide a controlled and gradual transition from large-scale exploration of the search space to precise local convergence. In the first phase of the algorithm, a hunger-based memory and movement mechanism is applied in order to promote population-based global exploration. This structure enables individuals to adapt their behavior not only according to their instantaneous fitness values but also based on their relative performance across successive iterations. Resetting the hunger level of individuals that reach the global best solution ensures that these solutions are preserved as reference points within the search process. Conversely, gradually increasing the hunger levels of individuals that fail to exhibit improvement encourages more aggressive and exploration-oriented movements. In this manner, the algorithm establishes a dynamic exploration–exploitation balance that mitigates the risk of premature convergence. The hunger level of each individual, denoted by $H_{i}\left(t\right)$, is defined in Equation (9) as a memory variable reflecting the relationship between the current fitness value and the global best solution.

(9) $$H_{i}\left(t\right)=\left\{\begin{array}{cc} 0, & \mathrm{if}\;fitness_{i}\left(t\right)=fitness_{best}\left(t\right),\\ H_{i}\left(t-1\right)+\alpha , & \mathrm{otherwise}. \end{array}\right.$$

Here, $H_{i}\left(t\right)$ represents the hunger value of the individual at time *t*, $fitness_{i}\left(t\right)$ denotes the fitness value of the current solution, and $fitness_{best}\left(t\right)$ indicates the global best fitness value at the corresponding iteration. The coefficient α controls the accumulation rate of hunger. Hunger levels are normalized to reflect relative differences among individuals and are utilized within probabilistic decision mechanisms as defined in Equation (10).

(10) $$s\left(H_{i}\right)=\frac{H_{i}}{\sum_{j=1}^{N}H_{j}}$$

Here, $s(H_{i})$ represents the hunger sensitivity ratio of the individual, while *N* denotes the population size. This ratio functions as an adaptive trigger that determines whether individuals adopt exploration-oriented or more conservative movement strategies. The position update process of individuals is modeled in Equation (11) by jointly considering hunger sensitivity, stochastic variables, and the proximity of solutions to the global optimum.

(11) $${\vec{x}}_{i}\left(t+1\right)=\left\{\begin{array}{cc} {\vec{x}}_{i}\left(t\right)\cdot \left(1+\mathrm{randn}(d)\right), & r_{1}<s\left(H_{i}\right),\\ {\vec{x}}_{best}\left(t\right)+\vec{R}\cdot W\cdot \left|{\vec{x}}_{best}\left(t\right)-{\vec{x}}_{i}\left(t\right)\right|, & r_{2}<E,\\ {\vec{x}}_{r1}\left(t\right)+\vec{R}\cdot W\cdot \left|{\vec{x}}_{r1}\left(t\right)-{\vec{x}}_{r2}\left(t\right)\right|, & \mathrm{otherwise}. \end{array}\right.$$

Here, ${\vec{x}}_{i}\left(t+1\right)$ denotes the position of the individual at the next iteration, $r_{1}$ and $r_{2}$ are random numbers generated in the interval [0,1], and $\vec{R}$ represents a random vector defined in the interval [−a,a]. The second movement behavior reflects an exploitation tendency that increases as the individual approaches the global best solution. The convergence incentive parameter *E* regulating this behavior is defined in Equation (12).

(12) $$E=\mathrm{sech}\left(|fitness_{i}-fitness_{best}|\right)$$

This formulation allows the parameter *E* to increase as the fitness value of an individual approaches the global optimum, thereby enabling the algorithm to exhibit solution-quality-sensitive exploitation behavior. Exploration intensity, on the other hand, is controlled through the weight coefficient *W*, which is adaptively determined according to the individual index in order to maintain population diversity, as shown in Equation (13).

(13) $$W=w_{1}\cdot e^{-\frac{N-i}{N}}+w_{2}$$

Although the HGS mechanism provides strong global exploration capability, a heavy-tailed Cauchy perturbation is integrated into this phase in order to reduce the risk of population stagnation in local minima. Unlike Gaussian perturbations, the heavy-tailed structure of the Cauchy distribution enables Lévy-like long-range jumps within the search space, allowing critical nucleotide positions to be modified simultaneously within a single iteration. In DNA data storage systems, the risk of cross-hybridization originates from the thermodynamic affinity of consecutive reverse-complement (RC) motifs within a sequence. Gaussian-based small and homogeneous variations tend to preserve such local motif structures and therefore fail to disrupt undesirable hybridization patterns. In contrast, the Cauchy operator modifies the sequential entropy of these consecutive regions in a single step, thereby destabilizing the Gibbs free energy (ΔG) associated with secondary structure formation. Furthermore, the adaptive decay of the coefficient β(t) enables the algorithm to break the molecular bonds of high-energy reverse-complement regions through functional mutations during the early stages of optimization, while preserving the obtained low RC similarity values and refining the local search process in later stages. This mechanism, which produces long-range jumps around the global best solution, is expressed in Equation (14).

(14) $${\vec{x}}_{cauchy}\left(t\right)=\mathrm{correct}\left({\vec{x}}_{best}\left(t\right)+{\vec{\delta }}_{cauchy}\cdot \beta \left(t\right)\right)$$

Here, ${\vec{\delta }}_{cauchy}$ denotes a random vector sampled from the Cauchy distribution, while β(t) represents the adaptive coefficient controlling the perturbation intensity. The function correct(·) is a boundary control mechanism that ensures that the generated candidate solutions remain within the integer search space [0,3], corresponding to the nucleotide representation, as defined in Equation (15).

(15) $$\beta \left(t\right)=0.015\cdot \left(1-\frac{t}{T_{HGS}}\right)$$

Following the global exploration phase, the algorithm transitions to a mathematical exploitation phase in order to refine the elite solutions obtained. During the second half of the iteration process ($t>0.5T_{max}$), the most successful 30% of the population are selected and transferred to a Runge–Kutta-inspired local refinement mechanism. The locally refined solution is obtained as shown in Equation (16).

(16) $${\vec{x}}_{RK}\left(t\right)={\vec{x}}_{best}\left(t\right)+\Delta \vec{x}\left(t\right)$$

Here, the displacement vector $\Delta \vec{x}\left(t\right)$ is defined in Equation (17).

(17) $$\Delta \vec{x}\left(t\right)=\vec{r}\cdot f_{refine}\left(t\right)\cdot \left({\vec{x}}_{best}\left(t\right)-{\vec{x}}_{rand}\left(t\right)\right)$$

In this formulation, $\vec{r}$ denotes a uniformly distributed random vector, while ${\vec{x}}_{rand}\left(t\right)$ represents the position of a randomly selected individual from the population. The refinement coefficient $f_{refine}\left(t\right)$ is defined in Equation (18) in such a way that local search steps gradually decrease as iterations progress.

(18) $$f_{refine}\left(t\right)=0.008\cdot e^{-3.0\frac{t}{T_{max}}}$$

This mechanism enables the algorithm to exhibit high-precision convergence behavior during the final stages of the optimization process. In parallel with this stage, an Adaptive Intensive Mutation layer is activated to eliminate persistent constraint violations in the nucleotide sequence. Within this surgical correction process, which is applied to 20% of the population, the mutation rate is adaptively updated according to Equation (19) as the iterations progress.

(19) $$Mutation\_Rate=0.02+0.03\cdot \left(\frac{t-0.5T_{max}}{0.5T_{max}}\right)$$

This dynamic structure increases the mutation intensity from 2% to 5% throughout the iteration process, thereby enabling high-dimensional constraint barriers to be eliminated in a precise and controlled manner.

### 4.3. Complexity Analysis and Pseudo-Code Representation of the Proposed Method

The computational cost and scalability analysis of the proposed TC-HUR algorithm reveals the theoretical limits of the hybrid architecture and its applicability to high-dimensional optimization problems. The overall time complexity of the algorithm is characterized as a linear function of the population size (*N*), the dimensionality of the solution space (*D*), and the total number of iterations (*T*). At the beginning of the process, the random initialization of the population and the computation of the initial fitness values incur a cost of O(N·D+N·f(D)), where f(D) represents the complexity of the fitness function incorporating DNA constraints. The HGS-based exploration phase, which constitutes the first half of the algorithm, requires $O(T_{HGS}\cdot N\cdot D)$ time at each iteration due to the update of hunger levels and the application of position update operators. The accompanying Cauchy Jump mechanism introduces only a marginal additional cost of $O(T_{HGS}\cdot D)$ since it is applied solely to the global best individual. In the second half of the iteration process, the Unified RUN phase is activated, which involves the refinement of the selected elite population using Runge–Kutta operators along with a concurrently executed intensive mutation layer. The computational cost of this phase remains bounded by $O(T_{RUN}\cdot N_{elite}\cdot D)$. The dominant cost component of the algorithm arises from the fitness function evaluations, where the entire population is assessed under biophysical constraints at each iteration, resulting in a complexity of O(T·N·f(D)). Consequently, the total time complexity of TC-HUR is determined as O(T·N·(D+f(D))). In terms of space complexity, the algorithm requires O(N·D) memory to store the population matrix (N×D), hunger vectors, and constraint violation records. This theoretical analysis demonstrates that the TC-HUR model provides linear scalability for high-dimensional DNA data storage problems and maintains a computational cost structure competitive with standard metaheuristics reported in the literature. The pseudo-code of the proposed method is presented in Algorithm 1.
**Algorithm 1** Tri-phase Cauchy-assisted HGS and Unified RUN (TC-HUR)**Require:** 
Population size *N*, maximum iterations *T*, dimension *D*, switching ratio SR=0.5, boundary ${\left[0,3\right]}^{D}$**Ensure:** 
Optimized DNA sequence ${\vec{x}}_{best}$1:**Initialization:** Generate initial population ${\vec{x}}_{i}\in Z^{D}$ and evaluate $Fitness({\vec{x}}_{i})$2:Set $T_{HGS}\leftarrow T\times SR$ and $T_{RUN}\leftarrow T-T_{HGS}$3:**while** t<T **do**4:    **if** $t\leq T_{HGS}$ **then**      ▹ **Phase 1: Global Exploration via Augmented HGS**5:        Update Hunger Levels $H_{i}\left(t\right)$ and weight *W* according to Equations (9) and (13)6:        Calculate convergence factor $E=\sec h(|f\left({\vec{x}}_{i}\right)-f\left({\vec{x}}_{best}\right)|)$7:        **Position Update:** Update ${\vec{x}}_{i}\left(t+1\right)$ using adaptive starvation weights *W*8:        **Cauchy Jump:** Apply ${\vec{x}}_{cauchy}\leftarrow \mathrm{correct}\left({\vec{x}}_{best}+{\vec{\delta }}_{cauchy}\cdot \beta \left(t\right)\right)$9:        **Elite Update: if** $f\left({\vec{x}}_{cauchy}\right)<f\left({\vec{x}}_{best}\right)$ **then** ${\vec{x}}_{best}\leftarrow {\vec{x}}_{cauchy}$10:    **else**         ▹ **Phase 2 & 3: Exploitation and Constraint Correction**11:        Select elite sub-population $N_{elite}=0.30N$12:        **RK-Refinement:** Compute displacement $\Delta \vec{x}=\vec{r}\cdot f_{refine}\left(t\right)\cdot \left({\vec{x}}_{best}-{\vec{x}}_{rand}\right)$13:        Perform local search via Runge–Kutta operators: ${\vec{x}}_{RK}\leftarrow {\vec{x}}_{best}+\Delta \vec{x}$14:        **Adaptive Intensive Mutation:** Update $Mut_{rate}$ from 0.02 to 0.0515:        Apply targeted mutation on ${\vec{x}}_{i}$ dimensions to eliminate $f_{HP}$ and $C_{RC}$ violations16:    **end if**17:    **Boundary Handling:** Map continuous solutions to discrete nucleotide space [0,3]∈{A,T,C,G}18:    **Fitness Evaluation:**19:    $f\left({\vec{x}}_{i}\right)\leftarrow \sum w_{obj}\cdot f_{obj}\left(\mathrm{GC},\;\mathrm{HP},\;\Delta G\right)+\sum w_{cons}\cdot C_{cons}\left(T_{m},\mathrm{RC},\;\mathrm{Ham}\right)$20:    Update global best ${\vec{x}}_{best}$ based on $f({\vec{x}}_{i})$21:    t←t+122:**end while**23:**return** ${\vec{x}}_{best}$ (Final Optimized DNA Sequence)

## 5. Conclusions

In this study, the TC-HUR hybrid algorithm developed to optimize the structural trade-off between biochemical constraints and information density in DNA-based data storage systems is presented. The proposed method enhances the global exploration capability of the HGS algorithm through the Cauchy jump operator, enabling it to escape local optimum traps in high-dimensional constrained spaces, while the RUN-based exploitation phase and the nucleotide-level Adaptive Intensive Mutation layer precisely refine constraint violations. Experimental results demonstrate that TC-HUR exhibits significant efficiency on the complex dataset consisting of 1853 nucleotides. According to the convergence analysis results, TC-HUR achieves an average fitness value of 3497.27, making it 2.5% more efficient than its closest competitor, the RUN algorithm, and 16.7% more efficient than the HGS algorithm. The algorithm becomes the only method capable of maintaining homopolymer length at the biologically ideal threshold of 3, while providing the lowest secondary structure risk with a reverse-complement (RC) value of 19.10%. Furthermore, the highest sequential diversity is achieved with a Hamming distance of 1415. Performance analyses under DNAStoralator noise channels reveal that TC-HUR obtains a NED value of 0.1290 under high error levels, demonstrating 12.2% higher efficiency than RUN and stabilizing substitution rates within the 0.03 range.

In future work, it is planned to extend this study in the following directions:

- Multi-objective Extension: Transforming the proposed framework into a multi-objective optimization architecture that directly minimizes inter-sequence cross-hybridization risk and secondary structure kinetics.
- Wet-lab Validation: Verifying the performance stability of sequences optimized by TC-HUR on real Illumina or Nanopore sequencing platforms by subjecting them to artificial synthesis processes.
- Hardware Acceleration and Scalability: Reducing computational costs through the adaptation of the algorithm to GPU-based parallel computing architectures and enabling scalable library design at the terabyte scale.
- Adaptive Error Modeling: Developing an adaptive constraint management layer that dynamically incorporates technology-specific error profiles (indel and substitution balance) associated with different synthesis technologies.

## Figures and Tables

**Table 1:** Control parameters of the algorithms used in the simulations and their optimization roles.

Algorithm	Parameter Configuration
GWO	a:2→0 (Linear)
PSO	$c_{1},c_{2}=2.05,\;w=0.4$
HGS	PUP=0.08, LH=10,000
RUN	a=20, f=0.1 (ESQ)
IAOA [35]	α=5, μ=0.5
TC-HUR Proposed	$\beta_{start}=0.015,f_{refine}=0.008$, $Mut_{rate}:0.02\to 0.05$ (Adaptive)

**Table 2:** PCR error regime configurations used in the DNAStoralator simulation.

Error Regime	PCR Cycle Range	Central Value	Biochemical Pressure
Low	10–100	50	Minimum noise level
Medium	50–400	200	Standard laboratory condition
High	100–1000	500	Maximum mutational load

**Table 3:** Comparative optimization and biophysical analysis of DNA sequences.

Algorithm	Mean Fitness	Std	Time (s)	GC (%)	$T_{m}$ (°C)	ΔG (kcal/mol)	Max HP	RC (%)	Hamming
GWO	4901.77	259.10	59.59	42.31	75.43	−2465.29	5	16.73	1392
PSO	4604.82	197.52	59.32	41.77	75.30	−2415.11	5	26.98	1412
HGS	4156.03	161.17	58.91	41.12	75.04	−2421.06	5	24.93	1384
RUN	3617.21	67.30	99.72	40.58	74.93	−2370.44	4	21.05	1419
IAOA	4943.48	157.02	58.47	46.84	77.38	−2511.68	5	24.61	1409
TC-HUR	3504.99	39.24	88.36	40.53	74.79	−2385.69	4	17.38	1425

**Table 4:** Comparative performance of the evaluated algorithms under different error regimes in the DNA storage channel.

Error Level	Algorithm	NED	Substitution Rate	Insertion Rate	Deletion Rate
Low	GWO	0.3732	0.1007	0.2149	0.0577
	PSO	0.4561	0.1272	0.2882	0.0407
	HGS	0.3163	0.0835	0.1921	0.0407
	RUN	0.1576	0.0344	0.0903	0.0329
	IAOA	0.4074	0.1085	0.2569	0.0419
	TC-HUR (Proposed)	0.1551	0.0302	0.0753	0.0496
Mid	GWO	0.3654	0.1030	0.2267	0.0357
	PSO	0.4549	0.1278	0.2923	0.0348
	HGS	0.3124	0.0858	0.1951	0.0315
	RUN	0.1492	0.0352	0.0918	0.0222
	IAOA	0.4063	0.1139	0.2605	0.0319
	TC-HUR (Proposed)	0.1333	0.0297	0.0795	0.0241
High	GWO	0.3633	0.1053	0.2296	0.0283
	PSO	0.4555	0.1305	0.2955	0.0294
	HGS	0.3108	0.0847	0.1965	0.0295
	RUN	0.1469	0.0351	0.0928	0.0190
	IAOA	0.4069	0.1127	0.2582	0.0360
	TC-HUR (Proposed)	0.1290	0.0300	0.0794	0.0195

**Table 5:** Homopolymer sensitivity analysis of the evaluated algorithms under different error regimes in the DNA storage channel.

Algorithm	Error Level	Avg Max HP	Avg HP Count	HP Indel (%)
GWO	Low	6.84	112.68	15.2
	Mid	6.48	116.38	14.9
	High	6.35	116.42	14.9
PSO	Low	6.61	111.81	14.1
	Mid	6.38	111.55	13.9
	High	6.27	112.79	14.0
HGS	Low	6.03	103.09	14.4
	Mid	5.86	103.12	14.2
	High	5.79	104.67	14.4
RUN	Low	5.19	82.13	14.5
	Mid	4.93	84.39	14.3
	High	4.96	84.94	14.7
IAOA	Low	6.29	99.90	13.6
	Mid	5.94	103.11	13.9
	High	6.18	102.64	13.9
TC-HUR (Proposed)	Low	5.68	76.21	13.5
	Mid	5.08	80.09	14.6
	High	4.89	80.19	13.8

**Table 6:** Context-Dependent Error Summary for Different Algorithms and Levels.

Algorithm	Level	GC→AT (%)	AT→GC (%)	HP Indel (%)	HP Sub (%)
GWO	Low	39.0	61.0	15.2	21.7
	Mid	39.3	60.7	14.9	21.5
	High	38.7	61.3	14.9	22.0
PSO	Low	37.5	62.5	14.1	20.9
	Mid	37.0	63.0	13.9	20.4
	High	36.9	63.1	14.0	20.4
HGS	Low	40.1	59.9	14.4	18.6
	Mid	39.4	60.6	14.2	18.4
	High	39.5	60.5	14.4	18.4
RUN	Low	39.6	60.4	14.5	12.3
	Mid	39.2	60.8	14.3	11.7
	High	39.1	60.9	14.7	11.6
IAOA	Low	46.7	53.3	13.6	15.7
	Mid	45.7	54.3	13.9	15.7
	High	46.9	53.1	13.9	15.6
TC-HUR (Proposed)	Low	38.4	61.6	13.5	14.2
	Mid	39.4	60.6	14.6	12.8
	High	40.8	59.2	13.8	12.7

## Data Availability

The original contributions presented in this study are included in the article. Further inquiries can be directed to the corresponding author.
